# The Cost of Providing Comprehensive HIV Services to Key Populations: An Analysis of the LINKAGES Program in Kenya and Malawi

**DOI:** 10.9745/GHSP-D-22-00538

**Published:** 2023-06-21

**Authors:** Marjorie Opuni, Jose Luis Figueroa, Jorge Eduardo Sanchez-Morales, Andrea Salas-Ortiz, Luz Edith Ochoa-Sanchez, Mariana Morales-Vazquez, Louis Masankha Banda, Alice Olawo, Spy Munthali, Julius Korir, Meghan DiCarlo, Navindra Persaud, Sergio Bautista-Arredondo

**Affiliations:** a Independent researcher, Geneva, Switzerland.; b Division of Health Economics and Health Systems Innovations, National Institute of Public Health, Cuernavaca, Mexico.; c Centre for Health Economics, University of York, York, United Kingdom.; d FHI 360, Lilongwe, Malawi.; e FHI 360, Nairobi, Kenya.; f University of Malawi, Zomba, Malawi.; g Kenyatta University, Nairobi, Kenya.; h FHI 360, Durham, NC, USA.

## Abstract

**Introduction:**

Timely data on HIV service costs are critical for estimating resource needs and allocating funding, but few data exist on the cost of HIV services for key populations (KPs) at higher risk of HIV infection in low- and middle-income countries. We aimed to estimate the total and per contact annual cost of providing comprehensive HIV services to KPs to inform planning and budgeting decisions.

**Methods:**

We collected cost data from the Linkages across the Continuum of HIV Services for Key Populations Affected by HIV (LINKAGES) program in Kenya and Malawi serving female and male sex workers, men who have sex with men, and transgender women. Data were collected prospectively for fiscal year (FY) 2019 and retrospectively for start-up activities conducted in FY2015 and FY2016. Data to estimate economic costs from the provider’s perspective were collected from LINKAGES headquarters, country offices, implementing partners (IPs), and drop-in centers (DICs). We used top-down and bottom-up cost estimation approaches.

**Results:**

Total economic costs for FY2019 were US$6,175,960 in Kenya and US$4,261,207 in Malawi. The proportion of costs incurred in IPs and DICs was 66% in Kenya and 42% in Malawi. The costliest program areas were clinical services, management, peer outreach, and monitoring and data use. Mean cost per contact was US$127 in Kenya and US$279 in Malawi, with a mean cost per contact in DICs and IPs of US$63 in Kenya and US$104 in Malawi.

**Conclusion:**

Actions undertaken above the service level in headquarters and country offices along with those conducted below the service level in communities, comprised important proportions of KP HIV service costs. The costs of pre-service population mapping and size estimation activities were not negligible. Costing studies that focus on the service level alone are likely to underestimate the costs of delivering HIV services to KPs.

## Abstract (precis)

A cost analysis of comprehensive HIV services for key populations at higher risk of infection in Kenya and Malawi showed that costs can be substantial at all program implementation levels, not just service delivery; all these costs should be considered during program planning.

## Teaser

**Key Findings:**

- Activities done at the headquarters and country office levels, as well as community outreach activities to engage key populations (KPs), comprised substantial proportions of total costs, suggesting that focusing on the costs of service delivery alone will likely underestimate the total costs of delivering HIV services to KPs.
- This analysis highlights the heterogeneity in costs and cost structure between Kenya and Malawi, illustrating the importance of differences in country contexts, needs, service delivery infrastructures, and capacities in influencing costs.
- These findings are consistent with the complexity of comprehensive KP HIV services; the levels of stigma, discrimination, and violence against KPs; and the support and oversight needs of implementing organizations.

**Key Implications:**

- Costing studies of HIV services for KPs should consider all activities conducted at all levels of program implementation.
- Resource needs assessments, budgeting exercises, and ultimately, funding allocations for KP HIV services in low-resource settings that only rely on service-level costs are based on flawed cost estimates.

## INTRODUCTION

Achieving HIV epidemic control by 2030 is a target of the Sustainable Development Goals,^1^ and despite remarkable progress, the international community is not on track to achieve this objective.^2^ Together with their partners, key populations (KPs) at higher risk of HIV infection—including men who have sex with men (MSM), sex workers, transgender women (TGW), people who inject drugs, and people in prisons—are estimated to make up 70% of new HIV infections globally.^2^ In many countries, KPs continue to be marginalized, stigmatized, and criminalized, and they are less likely to have access to HIV services.^3^ HIV epidemic control will only be achieved if countries around the world significantly increase the coverage of effective HIV services for KPs.^4^^,^^5^ To date, insufficient funding has been allocated to HIV services for KPs globally.^6^^,^^7^ For many countries, scaling up HIV services for KPs will require that additional financial resources be allocated to these services.

To allocate HIV resources efficiently and manage HIV programs effectively, funders and implementers require accurate and timely data on HIV service costs. There is a growing body of literature on the costs of HIV services for general populations in low- and middle-income countries.^8^ However, data on the costs of providing HIV services to KPs remain scarce.^8^^,^^9^ Furthermore, existing cost estimates for HIV services for KPs have focused on costs at the facility or service level.^10^ Most of these estimates fail to consider the costs of pre-service delivery start-up activities, interventions in the community to reach and engage KPs, and project management and oversight. Thus, resource needs assessments, budgeting exercises, and ultimately, funding allocations for HIV services for KPs in low-resource settings mostly rely on flawed cost estimates. We are aware of only 1 costing study of a comprehensive HIV program for female sex workers (FSWs), MSM, and TGW in India—the Avahan program—that included service-level costs, costs of interventions required to reach and engage KPs, and project management costs.^11^

To allocate HIV resources efficiently and manage HIV programs effectively, funders and implementers require accurate and timely data on HIV service costs.

The dearth of information on costs of comprehensive HIV services for KPs in low-resource settings is partly explained by the scarcity of well-documented HIV services for KPs in these locations and by their complexity.^10^^,^^12^^,^^13^ Five KP program characteristics need to be considered in the design of studies on KP HIV service costs to ensure the complete costing of these services. First, there are pre-service delivery activities, including population mapping and size estimation to identify the KPs in need of services in a geographic area, that are integral to the effective delivery of HIV services for KPs.^12^^,^^14^^,^^15^ Second, KP HIV services comprise both clinical services and structural interventions, such as interventions that seek to reduce stigma, discrimination, and violence against KPs.^16^^–^^18^ Third, besides activities undertaken at the service level, KP HIV services also consist of important interventions below and above the service level.^11^^,^^19^^,^^20^ KPs often require substantial outreach efforts conducted in the community to facilitate service utilization.^21^^–^^23^ Many KP programs include community-based approaches to service delivery, such as mobile services.^13^ Likewise, community organization staff and volunteers often need substantial support, including program management support, technical assistance, training, and oversight from individuals and institutions above the service level. Fourth, most KP services are provided through community-based organizations,^23^ funded from multiple sources,^24^ and the cadre of service providers varies and includes many temporary workers and community volunteers. Fifth, the availability and quality of data differ across organizations, making it difficult to capture program inputs and their costs, as well as program outputs.

The Linkages across the Continuum of HIV Services for Key Populations Affected by HIV (LINKAGES) program aimed to reduce HIV transmission among KPs and improve their enrollment and retention in care and treatment services in countries in Africa, Asia, and the Caribbean. The program was funded by the U.S. Agency for International Development through the U.S. President’s Emergency Plan for AIDS Relief from 2014 to 2021. FHI 360 administered LINKAGES in collaboration with Pact, IntraHealth International, and the University of North Carolina at Chapel Hill. In each country, operationalization of LINKAGES comprised an initial start-up phase consisting of pre-service delivery activities. Subsequently, a comprehensive package of KP HIV services was scaled up. Many of the activities scaled up as part of LINKAGES are now supported by the Meeting Targets and Maintaining Epidemic Control (EpiC) project funded by the U.S. Agency for International Development through the U.S. President’s Emergency Plan for AIDS Relief.

This study aimed to estimate the total and per contact annual cost of providing comprehensive HIV services to KPs in Kenya and Malawi to inform planning and budgeting decisions. We show cost estimates for the LINKAGES program in the 2 countries during U.S. Government fiscal year (FY) 2019 (October 1, 2018 to September 30, 2019). Because the activities of the start-up phase are indispensable to the delivery of effective comprehensive KP HIV services, we also present the estimates of the costs for pre-service delivery population mapping, size estimation, and program planning activities conducted in FY2015 and FY2016 in Malawi and FY2016 in Kenya. Unlike most existing work on costs of KP HIV services in low-resource settings, we derived cost estimates using a costing approach that considered all activities undertaken in the LINKAGES program at all levels of program implementation—at the service level, above the service level in headquarters and country offices, and below the service level in communities.

## METHODS

### Study Setting

Kenya and Malawi were purposively selected in consultation with program implementers. Both countries have generalized epidemics with concentrated sub-epidemics among KPs.^25^ In 2021, HIV prevalence in adults aged 15–49 years was estimated to be 4% in Kenya^26^ and 7.7% in Malawi.^27^ Recent HIV prevalence data for FSWs and MSM in Kenya are not available.^26^ In Malawi, the latest HIV prevalence data for FSWs and MSM were 49.9% (2020) and 12.9% (2020).^27^ In both countries, KPs face important structural barriers that increase their vulnerability to HIV infection and hinder their access to health services.^28^

### Program Description

LINKAGES program activities were executed at several implementation levels (Supplement Figure S1). High-level program guidance and technical assistance were provided by LINKAGES program headquarters. In each partner country, a LINKAGES office provided program management and technical support. Services were delivered to KPs by local community-based organizations, including KP-led organizations, referred to as implementing partners (IPs). The LINKAGES program provided IPs with funding, capacity-building, program guidance, and technical assistance to enable them to provide a minimum package of comprehensive HIV services to the KPs they served. Services were delivered in communities through outreach activities and at drop-in centers (DICs)—sites where KPs received HIV services, met with peers, and conducted social and community mobilization activities. The specific KPs served and the range of services offered varied somewhat by DIC. In Kenya, DICs provided services to FSWs, male sex workers, and MSM; in Malawi, DICs provided services to FSWs, MSM, and TGW.

The LINKAGES program comprised 7 core program areas: (1) engage KPs in population size estimation, mapping, and program planning; (2) KP empowerment and engagement; (3) structural interventions; (4) peer outreach; (5) clinical services; (6) program management; and (7) monitoring and data use. These core program areas were further divided into program elements, detailing the interventions implemented (Supplement Figure S2). All the programmatic work and technical assistance necessary for implementing comprehensive KP HIV services were organized along these program areas and elements. Global guidance informed the selection of services included in the LINKAGES service package.^13^^,^^29^^–^^32^ The cost-effectiveness of many of these interventions is well established,^33^^–^^37^ and the implementation of these services in combination rather than individually has been shown to increase efficiency and effectiveness.^7^^,^^38^

### Study Sample

The study sample reflected the multilevel LINKAGES program implementation structure. The sample comprised LINKAGES program headquarters; 2 LINKAGES country offices (1 per country); 18 IPs in Kenya and 2 in Malawi; and 30 DICs in Kenya and 15 in Malawi (Supplement Figure S1). In Kenya, we included all IPs and DICs that were part of the LINKAGES program in FY2019. In Malawi, we excluded 1 IP (and its DICs), given disagreements between FHI 360 and the IP at the time of the study, which ended in contract termination.

### Costing Frameworks

We developed costing frameworks for each country to capture the activities at all implementation levels and ensure complete costing of the LINKAGES program. The costing frameworks mapped activities and associated inputs and outputs to the 7 program areas and corresponding program elements. To capture pre-service delivery activities, we developed costing frameworks for the start-up years (FY2015 and FY2016 for Malawi and FY2016 for Kenya). To describe the ongoing activities undertaken at the service level in IPs and DICs and above the service level in headquarters and country offices, we developed costing frameworks for both countries for FY2019.

We developed costing frameworks for each country to capture the activities at all implementation levels and ensure complete costing of the LINKAGES program.

To complete the activities sections of the frameworks, we extracted each activity from country work plans, mapped it to the LINKAGES core program area and program element to which it contributed, and identified the level of program implementation: LINKAGES program headquarters and country offices, IP/DIC, and community. We defined start-up activities as those implemented early in the program’s life cycle that lay the groundwork for service delivery (i.e., activities implemented before service delivery started). These start-up activities included actions undertaken related to planning, mapping and population size estimation, staff recruitment, staff training, materials and systems development, infrastructure expansion, and the formulation of legal agreements. To complete the inputs sections of the frameworks, we extracted data on the resources required to conduct each activity from the work plans. To fill in the outputs section of the frameworks, we extracted outputs from indicator matrices incorporated in the country work plans. We mapped each output to the relevant LINKAGES program core area and program element and to the level of program implementation at which the output was produced. The frameworks were reviewed and validated by staff in LINKAGES program headquarters and in the LINKAGES country offices of Kenya and Malawi.

### Ethical Clearance

The ethical review board of the National Institute of Public Health, Mexico, approved the study (CI: 1554). Informed consent was not required for this study, which did not involve human subjects research.

### Data Collection

This costing study was implemented following the guidelines of the Global Health Cost Consortium.^39^ We collected data retrospectively for the start-up years (FY2015 and FY2016 for Malawi and FY2016 for Kenya) and prospectively for FY2019. Data were collected from LINKAGES country offices, IPs, and DICs using a standardized set of Excel-based data collection tools. These tools were tested and fine-tuned during the collection of retrospective data for FY2018. Cost data were obtained from financial reports, payroll records, program manager reports, facility consumption data reports, program expense files, and asset registers. Additional data from headquarters and country offices were extracted from expenditure records provided by LINKAGES program headquarters. Data on quantities and prices were collected for the following input categories: clinical supplies, staff, peer workers, transportation, utilities and operations, external services, equipment, and training (Supplement Table S1). We collected monthly data on inputs irrespective of funding source. Corresponding output data were obtained from databases in IPs and DICs. We collected information on time spent on each of the 7 LINKAGES program areas from staff in country offices, IPs, and DICs.

### Cost Estimation

Costs were estimated from the provider’s perspective (i.e., the LINKAGES program). We estimated economic costs, which consider the value of all resources used, including those for which there was no financial transaction, such as donated male and female condoms. Annual costs were estimated for FY2019. Our cost estimates also include start-up costs. Start-up and capital costs were annualized using a discount rate of 3% and assumed to have a useful life of 10 years.^39^ We used a combination of top-down and bottom-up costing approaches.^11^ Top-down methods were used to estimate headquarters, country office, and IP costs and allocate them to DICs. Bottom-up methods were used to measure the quantities and prices of all inputs used to produce services in DICs.

We calculated the above service level (headquarters and country office) and pre-service (start-up) costs and allocated them across DICs (Supplement Table S2). LINKAGES headquarters and country office costs for FY2019 were distributed equally across DICs, assuming that all DICs received the same level of support from headquarters and country office staff. The start-up costs for FY2019 were apportioned equally across DICs.^39^

We proceeded as follows for service level (IP and DIC) costs. For the 13 IPs in the sample with only 1 DIC, we allocated all IP costs to the corresponding DIC. For the 7 IPs with multiple DICs, the allocation approach used depended on the input. We distributed IP costs for staff, other recurrent inputs, and equipment proportionally across DICs. Transportation and training costs, which were available only at the IP level, were distributed across DICs based on service level staff time weights (Supplement Table S2). To estimate the costs at the DIC level for clinical supplies, staff, peer workers, other recurrent inputs, and equipment, we multiplied the quantity of inputs used with their prices, which were collected through our data collection tool. Total LINKAGES program costs per DIC were obtained by summing the headquarters and country office allocations to DICs with IP and DIC costs.

To reflect LINKAGES program implementation, we disaggregated total DIC costs by core program area (Box). We used different allocation approaches to distribute the above service level, pre-service level (start-up), and service level costs in each DIC to the 7 LINKAGES program areas (Supplement Table S3). Program area staff time weights were used to allocate headquarters, country office, and start-up costs to the 7 program areas. The costs of IP/DIC equipment and other recurrent inputs were also allocated to the 7 program areas based on program area staff time weights. DIC costs for clinical supplies, staff, peer workers, and IP/DIC costs for transportation and training were apportioned to the 7 program areas using the detailed information on the share of these inputs allocated to program areas collected through our data collection instrument.

BOX LINKAGES Core Program Areas

1. Engage KPs in population size estimation, mapping, and program planning
2. KP empowerment and engagement
3. Structural interventions
4. Peer outreach
5. Clinical services, including condom and lubricant promotion and distribution, sexually transmitted infection services, pre-exposure prophylaxis, post-exposure prophylaxis, HIV testing services, antiretroviral therapy, sexual and reproductive health services, and management of sexual violence
6. Program management
7. Monitoring and data use

Unit cost per contact was calculated for each DIC by dividing DIC total costs by the total number of contacts made by each DIC in FY2019. A contact was defined as an individual contacted by the LINKAGES program through individual or small group HIV prevention interventions (with some individuals reached more than once). No data were available on unique individual contacts made.

All costs are presented in 2019 U.S. dollars (US$). Country costs for FY2019 were converted from local currencies to U.S. dollars using midyear exchange rates for 2019 (Kenya: 102.01 Kenyan shillings; Malawi: 739.46 Malawian kwacha). Headquarters costs were reported in 2019 U.S. dollars. Start-up costs for FY2015 and FY2016, which were reported in U.S. dollars, were inflated to 2019 U.S. dollars.

## RESULTS

### Start-Up Costs

**FIGURE 1:**
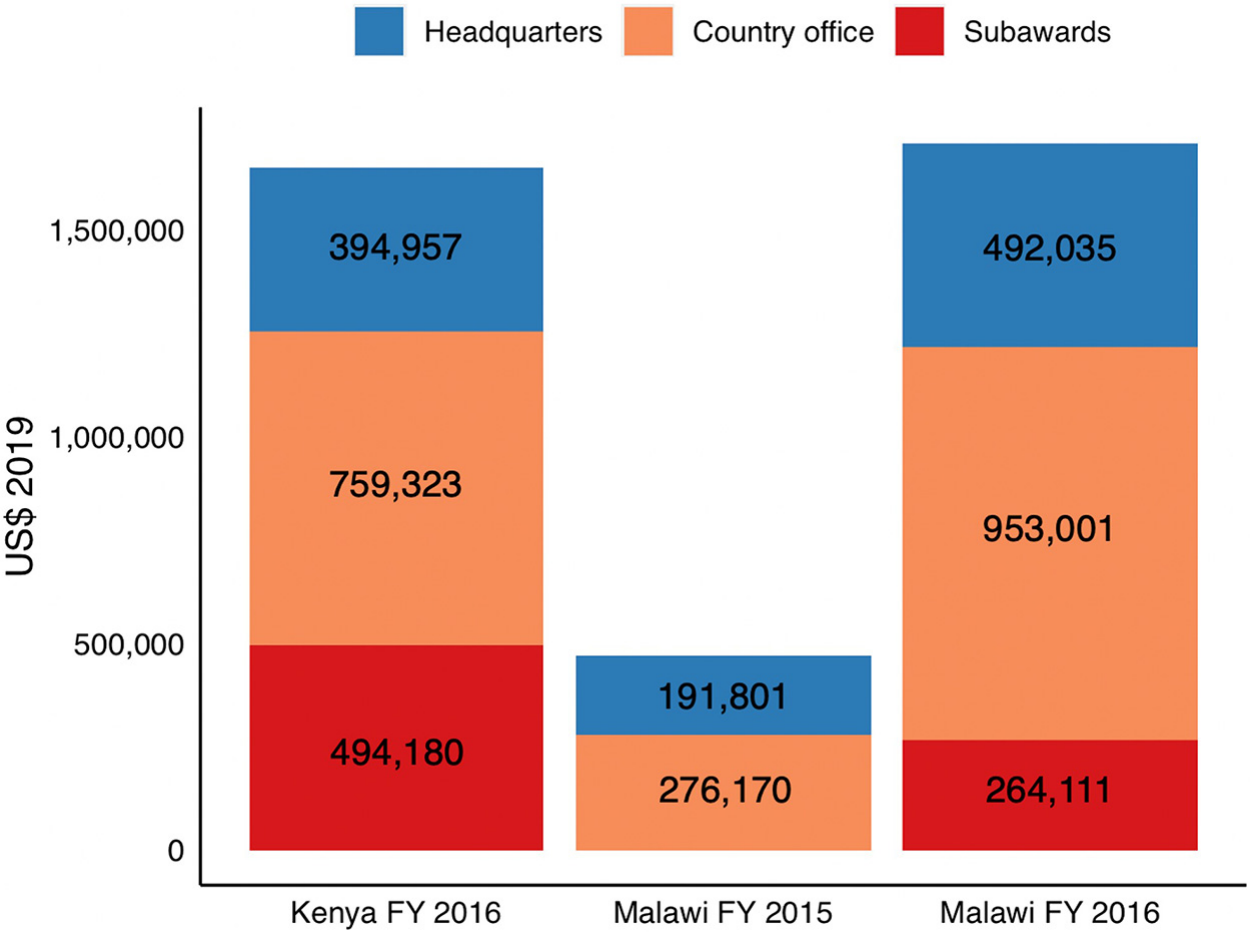
LINKAGES Program Start-Up Costs by Country and Program Implementation Level, FY2015 and FY2016^a^ Abbreviations: FY, fiscal year; LINKAGES, Linkages across the Continuum of HIV Services for Key Populations Affected by HIV; US$, U.S. dollars. ^a^Headquarters refers to LINKAGES program headquarters, country office refers to LINKAGES program country office, and subawards refers to grants given to implementing partners in countries. FY 2015 refers to U.S. Government fiscal year from October 1, 2014 to September 30, 2015. FY2016 refers to U.S. Government fiscal year from October 1, 2015 to September 30, 2016.

The total economic cost of LINKAGES program start-up activities in Kenya was US$1,648,460, with 24% spent at headquarters, 46% spent at the country office, and 30% disbursed as subawards to IPs in the country (Figure 1). In Malawi, the total cost of start-up activities was US$2,177,118, with 31% spent at headquarters, 57% spent at the country office, and 12% paid as subawards to IPs.

### Total Costs

The total economic cost of the LINKAGES program in FY2019 in Kenya was US$6,175,960, with 34% spent above the service level at headquarters (12%) and at the country office (22%) and 66% spent at the service level in IPs and DICs (Table 1). In Malawi, the total economic cost of the program in FY2019 was US$4,261,207, with 58% spent at headquarters (21%) and the country office (37%) and 42% spent at IPs and DICs.

**TABLE 1.** Total Economic Costs of LINKAGES Program by Country, Program Area, and Program Implementation Level, FY2019^a^

**Program Areas**	**Kenya Total Costs, US$**				**Malawi Total Costs, US$**			
	**HQ**	**CO**	**IP/DIC**	**Total (%)**	**HQ**	**CO**	**IP/DIC**	**Total (%)**
KP size estimation^b^	35,576	78,795	90,831	205,203 (3)	36,617	135,136	72,754	244,507 (6)
KP empowerment^c^	30,059	151,713	85,432	267,205 (4)	35,969	99,645	71,052	206,666 (5)
Structural interventions	31,341	98,573	87,804	217,719 (4)	25,150	43,244	48,405	116,799 (3)
Peer outreach	86,464	72,148	671,587	830,198 (13)	95,771	242,535	318,434	656,740 (15)
Clinical services	221,888	59,523	2,147,190	2,428,601 (39)	307,642	228,193	672,074	1,207,909 (28)
Management	135,519	670,179	549,243	1,354,940 (22)	185,267	407,268	337,930	930,464 (22)
Monitoring and data use	135,088	161,060	381,860	678,008 (11)	110,439	237,615	214,760	562,814 (13)
Start-up costs	46,581	89,280	58,226	194,086 (3)	107,298	187,772	40,237	335,307 (8)
Total (%)^d^	722,516 (12)	1,381,272 (22)	4,072,173 (66)	6,175,960 (100)	904,153 (21)	1,581,409 (37)	1,775,645 (42)	4,261,207 (100)

Abbreviations: CO, country office; DIC, drop-in center; FY, fiscal year; HQ, headquarters; IP, implementing partner; KP, key population; LINKAGES, Linkages across the Continuum of HIV Services for Key Populations Affected by HIV; US$, U.S. dollars.

a U.S. Government fiscal year from October 1, 2018 to September 30, 2019.

b Engaging KPs in population size estimation, mapping, and program planning.

c KP empowerment and engagement.

d Numbers may not add up due to rounding.

In terms of the costs per LINKAGES program area, clinical services, management, peer outreach, and monitoring and data use were the costliest program areas in Kenya. Above the service level, the costliest program areas were management, monitoring and data use, and clinical services. At the service level, clinical services, peer outreach, and management were the program areas that incurred the highest costs.

The costliest program areas in both countries were clinical services, management, peer outreach, and monitoring and data use.

The distributions were similar in Malawi, where overall, clinical services, management, peer outreach, and monitoring and data use were the costliest program areas. Above the service level, management, clinical services, peer outreach, and monitoring and data use incurred the highest costs compared to clinical services, management, peer outreach, and monitoring and data use at the service level.

Table 2 shows the breakdown of total costs at IPs and DICs only in Kenya and Malawi by input category (this breakdown is not available for costs at the headquarters and country office levels). Of the US$4,013,947 spent at the IPs and DICs in Kenya, clinical supplies, staff, other recurrent, and peer workers were the inputs with the highest costs. Of the US$1,735,407 spent at the IPs and DICs in Malawi, staff, clinical supplies, peer workers, and other recurrent were the inputs with the highest costs. For further breakdown of the clinical supply costs in each country, see Supplement Table S4. Condoms and lubricants and ART drugs were the 2 clinical inputs incurring the highest costs in both countries.

**TABLE 2.** Total Economic Costs of LINKAGES Program per Type of Input at the IP/DIC Level, FY2019^a^

**Inputs**	**Kenya Total Costs, US$ (%)**	**Malawi Total Costs, US$ (%)**
Clinical supplies	1,644,707 (41)	262,096 (15)
Staff	1,142,154 (28)	1,058,690 (61)
Peer workers	447,426 (11)	183,757 (11)
Transportation	66,466 (2)	6,957 (0)^b^
Other recurrent	625,826 (16)	166,637 (10)
Equipment	26,341 (1)	16,937 (1)
Training	61,026 (2)	40,333 (2)
Total IP/DIC (%)^c^	4,013,947 (100)	1,735,407 (100)

Abbreviations: DIC, drop-in center; FY, fiscal year; IP, implementing partner; LINKAGES, Linkages across the Continuum of HIV Services for Key Populations Affected by HIV; US$, U.S. dollars.

a U.S. Government fiscal year from October 1, 2018 to September 30, 2019.

b Amount equal to 0.4%.

c Numbers may not add up due to rounding.

**FIGURE 2:**
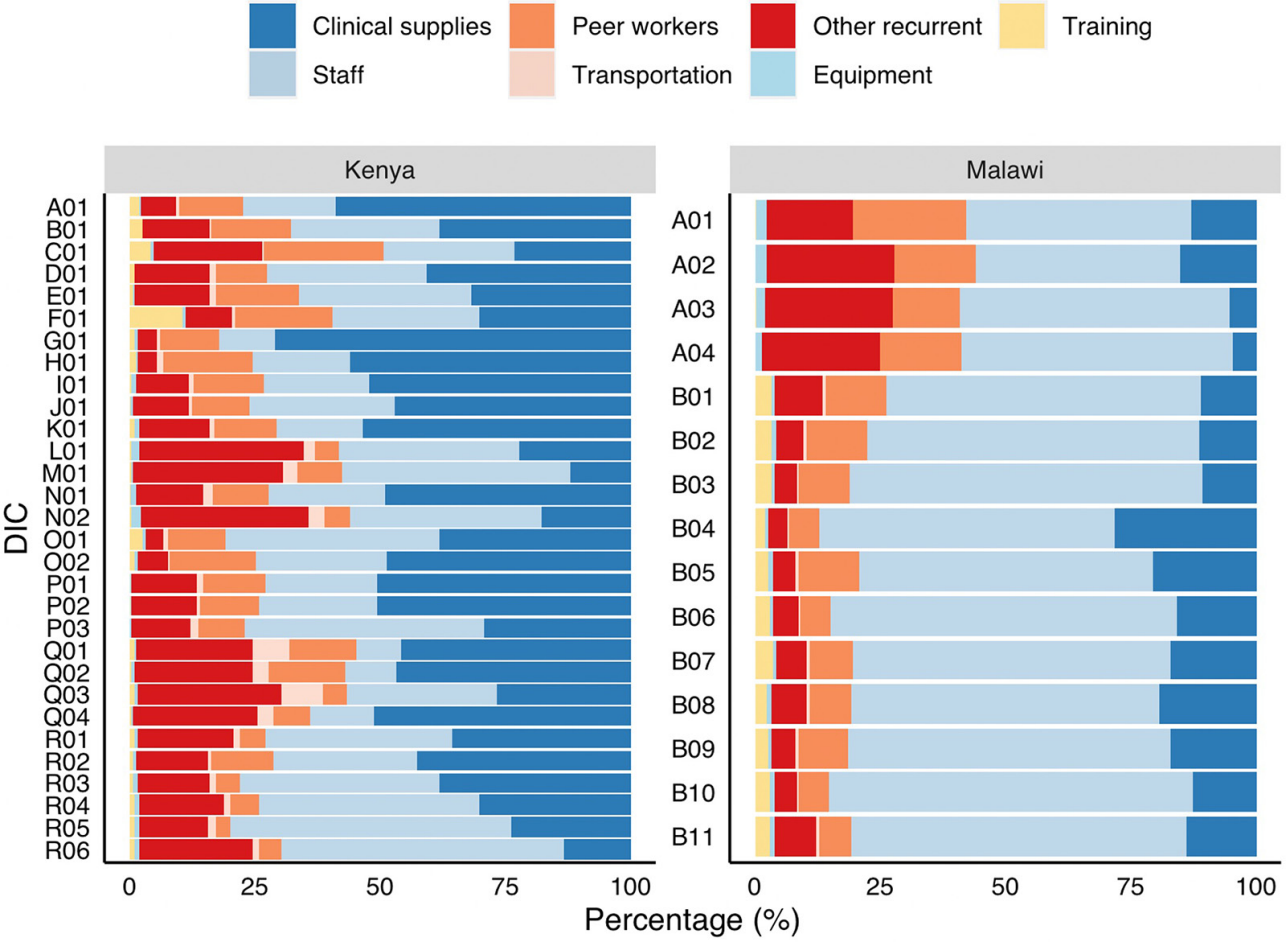
Total Economic Costs of LINKAGES Program by Country, Type of Input, and DIC, FY2019^a^ Abbreviations: DIC, drop-in center; FY, fiscal year; IP, implementing partner; LINKAGES, Linkages across the Continuum of HIV Services for Key Populations Affected by HIV. ^a^ Each letter number combination on the y-axis represents a DIC with the letters representing the associated IP. FY2019 refers to U.S. Government fiscal year from October 1, 2018 to September 30, 2019.

Figure 2 displays the breakdown of total costs by input category for each of the DICs in the sample, with DICs clustered by their managing IPs. Figure 2 shows substantial variation across DICs. In Kenya, for example, clinical supplies represented between 12% and 71% of total costs, whereas staff comprised between 9% and 56% of costs. In Malawi, the same categories ranged from 5% to 28% and 51% to 73%, respectively. Figure 2 also highlights substantial differences in input cost profiles across DICs associated with the same IP, especially in Kenya. For instance, in DICs associated with IPs “Q” and “R,” clinical supplies ranged from 27% to 51% and 13% to 43% of costs, respectively.

### Unit Costs

Table 3 presents the number of contacts and the unit costs per contact in Kenya and Malawi in FY2019. DICs were on average larger (in terms of contacts) in Kenya than in Malawi. The mean total unit cost per contact was US$127 in Kenya and US$279 in Malawi. The mean unit cost above the service level at headquarters and the country office was US$44 in Kenya and US$161 in Malawi, while the mean unit cost at the service level at IPs and DICs was US$63 in Kenya and US$104 in Malawi. Table 3 also shows the variation in unit cost per contact within countries. For further breakdown of the unit costs per contact by KP served in each country, see Supplement Table S5. Unfortunately, our sample size does not allow for statistical analysis of the differences in cost across the different kinds of DICs in each country. Though mean unit costs in DICs serving MSM and MSM/TGW may be higher, this is likely driven in large part by the smaller numbers of persons contacted compared to DICs serving other populations.

**TABLE 3.** Number of LINKAGES Program Contacts and Costs per Contact by Country and Service Level, FY2019^a^

	**Kenya**	**Malawi**
Number of DICs	30	15
Number of contacts^b^		
Mean (SD)	6,347 (12,205)	1,184 (436)
Range	422–51,823	570–2,159
Median (IQR)	2,326 (2,461)	1,140 (413)
**Cost per contact,^b^ US$**		
Headquarters and country office level		
Mean (SD)	44 (39)	161 (59)
Range	1–171	78–295
Median (IQR)	31 (41)	148 (58)
Implementing partner/drop-in center level		
Mean (SD)	63 (55)	104 (23)
Range	5–311	79–151
Median (IQR)	54 (39)	94 (38)
Total		
Mean (SD)	127 (102)	279 (79)
Range	10–554	180–455
Median (IQR)	119 (75)	255 (116)

Abbreviations: DIC, drop-in center; FY, fiscal year; IQR, interquartile range; LINKAGES, Linkages across the Continuum of HIV Services for Key Populations Affected by HIV; SD, standard deviation; US$, U.S. dollars.

a U.S. Government fiscal year from October 1, 2018 to September 30, 2019.

b Mean (SD), range, and median (IQR) calculated across all DICs in the sample for each country.

## DISCUSSION

In this descriptive analysis of the costs of KP HIV services delivered by the LINKAGES program in Kenya and Malawi in FY2019, we considered all program elements and all levels of program implementation. Our estimates of total economic costs for both countries underscore that (1) actions undertaken above the service level at headquarters and the country office and work done in the community to reach and engage KPs comprise important proportions of costs and (2) the costs of pre-service activities are not negligible. In Kenya, above service level costs made up 34% of costs, while in Malawi, they constituted 58%. Whereas clinical services comprised 39% of total costs in Kenya and 28% in Malawi, outreach activities made up 13% of costs in Kenya and 15% in Malawi. The costs of start-up activities prorated over 10 years made up 3% of FY2019 costs in Kenya and 8% in Malawi. Our findings suggest that studies focusing only on service-level costs are likely to underestimate the costs of delivering HIV services to KPs. Our findings are consistent with the complexity of comprehensive HIV services for KPs; the levels of stigma, discrimination, and violence against KPs in Kenya and Malawi further adding to this complexity; and the support needed by implementing organizations to operate effectively, including program management support, technical assistance, training, and oversight from above the service level.

Our findings suggest that studies focusing on service-level costs are likely to underestimate the costs of delivering HIV services to KPs.

Our analysis also highlights the heterogeneity in costs and cost structure between the 2 countries. Mean unit cost per contact was lower in Kenya than it was in Malawi. In general, organizations in Kenya delivered significantly more services than those in Malawi, and these differences in service volumes likely play an important role in cost differences between the 2 countries. In addition, some of the difference in cost was because of the higher start-up, headquarters, and country office costs in Malawi. Country contexts and needs differ, and the nature and capacity of community-based organizations also vary within and between countries. The LINKAGES program established the DICs operating in Malawi as none existed previously, while in Kenya, the program worked with already existing DICs. Therefore, service delivery sites in Kenya were older and more robust than those in Malawi and likely required less headquarter and country office support to provide services. Summaries of LINKAGES program achievements in Kenya and Malawi also suggest that the program in Malawi may have had a larger portfolio of above service level activities playing key roles in national HIV policy and guideline development.^40^^,^^41^

We also found differences in the costs and cost structure across DICs within each country and across DICs associated with the same IP. Some of the difference may be because of variations in the KPs served and services provided, but much of the heterogeneity in costs across DICs is likely associated with differences in the volume of contacts. In addition, variation in costs across DICs is also likely to represent differences in the efficiency of service delivery. We will explore the determinants of cost differences across DICs in future work.

We are aware of only 1 other study that examined the costs incurred by a comprehensive HIV program for KPs at the various program implementation levels. A cost analysis of the Avahan program in India that provided services to FSWs, MSM, and TGW also considered the full above service level costs of program implementation.^11^ Comparing the mean unit cost per contact we found for Kenya (US$127) and Malawi (US$279) to those estimated for the Avahan program is difficult. Input costs vary over time and across countries, the outputs used in Avahan (person reached and monthly contact) differed from those in this study, costing estimation methods used in the 2 studies were not the same, and the range of services delivered in both programs differed (the clinical services provided in the Avahan program were more limited in scope; most notably the Avahan program did not provide antiretroviral therapy). However, it is interesting to compare the distributions of costs across service levels in this study to those found in the Avahan costing study. The multi-year analysis of comprehensive KP HIV services in a different epidemiological setting showed that though this proportion tended to decrease with time, the proportion of above service level costs was always more than half of total costs. As the authors of the Avahan cost analysis explain, assessing the appropriate level of these costs is complex because, besides program management, these costs are used to support and develop the capacity of service providers in various ways.^11^ Such an assessment is further complicated by the fact that very little information exists on above service level costs in global health programs—most costing studies on health interventions exclude these costs as data on them are often difficult to obtain.^39^

### Strengths and Limitations

A major strength of this study is that our analysis was a comprehensive evaluation of the costs of HIV services for KPs. Using detailed costing frameworks, we considered all activities undertaken in the LINKAGES program, including the start-up activities essential to the effective implementation of a comprehensive KP HIV program that were conducted before service delivery began. We also considered all levels of LINKAGES program implementation. This study is also among only a few studies conducted on the costs of HIV services for KPs in sub-Saharan Africa in recent years.^42^^,^^43^

Several limitations should be kept in mind when considering our findings. We used routine monitoring data to capture information on outputs, and the level of detail, quality, and completeness of these data varied. Although the LINKAGES program had a reasonably robust system for data reporting and verification, under-reporting of results would cause an overestimation of unit costs, and over-reporting would lead to an underestimation of costs. Documentation of donated goods also varied across IPs and DICs, and although we tried to capture these goods adequately, some misreporting of in-kind contributions is possible. While detailed micro-costing methods were used to assess costs in IPs and DICs, we used top-down methods to estimate above service level costs and allocate them to DICs and program areas. The allocation of above service level costs was based on assumptions and weights based on staff effort, but detailed time allocation information was not collected from headquarters staff.

## CONCLUSION

This study of the costs of comprehensive HIV services for KPs in Kenya and Malawi underscores the need for costing studies of HIV services for KPs to consider all activities provided at all levels of program implementation. Policymakers, planners, and implementers working on resource needs estimates, budgeting exercises, and funding allocations for HIV services for KPs should know that non-service level costs associated with KP HIV service delivery are considerable and ensure that these are considered.

## Supplementary Material

GHSP-D-22-00538-supplement.pdf

## Notes

**Peer Reviewed**

**First published online:** June 1, 2023.
