# Supplementary material for: The Cost of Providing Comprehensive HIV Services to Key Populations: An Analysis of the LINKAGES Program in Kenya and Malawi
Source: Glob Health Sci Pract. 2023 Jun 21;11(3):e2200538. doi: 10.9745/GHSP-D-22-00538 (PMC10285728; doi:10.9745/GHSP-D-22-00538)
Supplement: GHSP-D-22-00538-supplement.pdf [file GHSP-D-22-00538-supplement.pdf]

Supplement to: Opuni M, Figueroa JL, Sanchez-Morales JE, et al. The cost of providing comprehensive HIV services to key populations: an analysis of the LINKAGES program in Kenya and Malawi. *Glob Health Sci Pract*. 2023;11(3):e2200538. https://doi.org/10.9745/GHSP-D-22-00538

SUPPLEMENT

Supplement Figure S1. LINKAGES program implementation levels and sample size by level

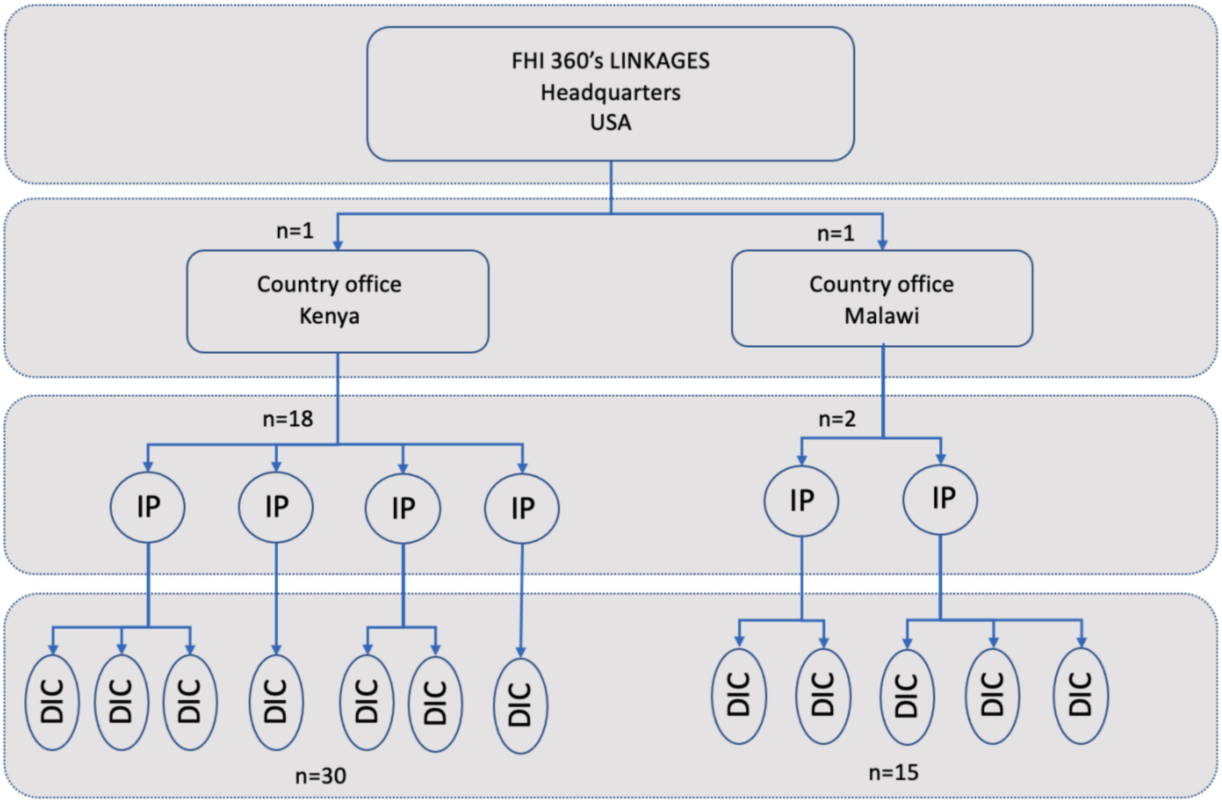

Notes: *LINKAGES* Linkages Across the Continuum of HIV Services for Key Populations Affected by HIV, *IP* Implementing partner, *DIC*, Drop-in center, *n* number of offices/organizations/facilities.

Supplement to: Opuni M, Figueroa JL, Sanchez-Morales JE, et al. The cost of providing comprehensive HIV services to key populations: an analysis of the LINKAGES program in Kenya and Malawi. *Glob Health Sci Pract*. 2023;11(3):e2200538. https://doi.org/10.9745/GHSP-D-22-00538

Supplement Figure S2. LINKAGES program areas and elements

Engage Key Populations in Population Size Estimation, Mapping, and Program Planning

1 National-level population size estimation and mapping
2 Local-level population size estimation and mapping
3 Hotspot-level population size estimation and mapping
4 Plan the program using mapping and size estimation data

Structural Interventions

1 Identify, design, and implement strategies to prevent and respond to violence against key population members
2 Develop strategies for reducing stigma in health-care settings

Monitoring and data use

1 Develop or adapt data-collection tools
2 Ensure the quality of data collection, analysis, and reporting
3 Regularly review and analyze data and use for programming

Key Population Empowerment and Engagement in Programs

1 Develop staffing of programs and teams by key population members
2 Establish drop-in centers
3 Support key population groups through capacity development and organizational strengthening
4 Foster oversight of clinical services and other services by the key population community

Peer Outreach

1 Map or validate key populations and set targets for outreach
2 Develop or adapt micro-planning tools
Recruit peer outreach workers
3 Train peer outreach workers
Implement and manage peer outreach
4 Provide advanced training and support for professional development
5 Support retention in care of HIV-positive key population members
6 Expand outreach to key population members through Enhanced Peer Mobilization (optional)

Program Management

1 Contract, hire, and train staff
2 Establish and implement policies and procedures on data safety, confidentiality, and ethics
3 Establish systems for supportive supervision and technical support

Clinical services

General considerations for establishing and providing clinical services:
1 Assess current services and the service needs of key populations
2 Organize effective, high-quality, available, and accessible services
3 Organize referral systems and track referrals

Considerations for specific clinical services:
4 Condom and lubricant promotion
5 STI services
6 Pre-exposure prophylaxis (PrEP)
7 Post-exposure prophylaxis (PEP)
8 HIV testing services (HTS)
9 Antiretroviral therapy (ART)
10 Prevention, screening, and management of common infections and co-infections
11 Harm reduction for people who inject drugs
12 Other drug and alcohol dependence
13 Sexual and reproductive health services, including family planning
14 Management of sexual violence
15 Mental-health care

Notes: Each program area is contained in a colored rectangle. Associated program elements are listed below each program area. (Source: FHI 360. LINKAGES Kenya FY18 Implementation Plan. 2017).

Supplement to: Opuni M, Figueroa JL, Sanchez-Morales JE, et al. The cost of providing comprehensive HIV services to key populations: an analysis of the LINKAGES program in Kenya and Malawi. *Glob Health Sci Pract*. 2023;11(3):e2200538. https://doi.org/10.9745/GHSP-D-22-00538

Supplement Table S1. Cost categories and sub-categories included in the LINKAGES costing study

Cost category	Sub-categories
**Clinical supplies**	Male condoms, female condoms, lubricants, STI treatment drugs, PEP drugs, PrEP drugs, ART drugs, family planning commodities, HIV and STI test
**Staff**	Monthly salary, total benefits package, medical insurance and worker compensation, life insurance, social security, pension fund, overtime pay, severance, paid leave, allowances, accommodation, relocation, benefits, etc.
**Peer workers**	Monthly payment (peer educators, peer navigators, outreach workers)
**Transportation**	Staff per diem, public transportation, rent of vehicles, fuel, vehicle maintenance, vehicle insurance, flights, general transportation, outreach transportation, supervision transportation
**Other recurrent (Utilities, operations, and external services)**	Electricity consumption, water consumption, diesel for generator, oil/paraffin/kerosene, internet connection, telephone service, rent, building maintenance, building insurance, general office supplies, security services
**Equipment**	Type of item bought (computing equipment, cabinets, chairs, desks, etc.)
**Training**	HIV care & treatment training, outreach workers training, peer educator training, annual review meetings, community advisory meetings, staff training, etc.

Notes: *STI* Sexually transmitted infections, *PEP* Post-Exposure Prophylaxis, *PrEP*, Pre-Exposure Prophylaxis, *ART*, Antiretroviral therapy.

Supplement to: Opuni M, Figueroa JL, Sanchez-Morales JE, et al. The cost of providing comprehensive HIV services to key populations: an analysis of the LINKAGES program in Kenya and Malawi. *Glob Health Sci Pract*. 2023;11(3):e2200538. https://doi.org/10.9745/GHSP-D-22-00538

Supplement Table S2. Methods used to estimate total LINKAGES program cost per DIC

- Above facility costs (headquarters, country office, implementing partners, and start up) were distributed to individual DICs (top-down approach).
- Bottom-up micro costing methods were used to measure the quantities and prices of inputs used to produce services in DICs.

<table>
<tr><th></th><th colspan="2">Allocation factors</th><th></th></tr>
<tr><th>Input description</th><th>DICs overseen by IP managing only one DIC</th><th>DICs overseen by IP managing more than one DIC</th><th></th></tr>
<tr><td>**Startup**</td><td></td><td></td><td rowspan="10">Top-down approach</td></tr>
<tr><td>Startup (expenditures at headquarters and country levels and subawards with no breakdown by input type)</td><td>Total cost was equally distributed across DICs</td><td>Total cost was equally distributed across DICs</td></tr>
<tr><td>**Implementation**</td><td></td><td></td></tr>
<tr><td>*Headquarters*</td><td></td><td></td></tr>
<tr><td>Program expenditures at HQ level (staff and indirect)</td><td>Total cost was equally distributed across DICs</td><td>Total cost was equally distributed across DICs</td></tr>
<tr><td>*Country office*</td><td></td><td></td></tr>
<tr><td>Program expenditures at CO level (staff, recurrent, external services, travel, tax, training, capital)</td><td>Total cost was equally distributed across DICs</td><td>Total cost was equally distributed across DICs</td></tr>
<tr><td>*Implementing partner (main office)*</td><td></td><td></td></tr>
<tr><td>Staff, utilities, external services, equipment</td><td>All costs allocated to corresponding DIC</td><td>Allocated proportionally to DICs overseen by IP</td></tr>
<tr><td>Transportation and training</td><td>All costs allocated to corresponding DIC</td><td>Allocated based on DIC staff time weights#</td></tr>
<tr><td>*DIC level costs*</td><td></td><td></td><td></td></tr>
<tr><td>Staff, peer workers, clinical supplies, utilities, external services, equipment, transportation and training</td><td colspan="2">Price was multiplied by quantity of each reported input and added to obtain the totals</td><td>Bottom-up approach</td></tr>
</table>

Notes: *CO* country office, *DIC* drop-in center, *HQ* headquarters, *IP* implementing partner

#$SLW_k = \frac{\sum_{k=1}^{n}\sum_{i=1}^{n} TD_{I,k,IP}}{\sum_{IP=1}^{n}\sum_{k=1}^{n}\sum_{i=1}^{n} TD_{i,k,IP}}$

where SLW =service level staff time weight, k=drop-in center, i=individual, IP=implementing partner, TD=time allocation

Supplement to: Opuni M, Figueroa JL, Sanchez-Morales JE, et al. The cost of providing comprehensive HIV services to key populations: an analysis of the LINKAGES program in Kenya and Malawi. *Glob Health Sci Pract*. 2023;11(3):e2200538. https://doi.org/10.9745/GHSP-D-22-00538

Supplement Table S3. Approach used to estimate costs per LINKAGES program area

Input description	Allocation factors
Known program area	
Clinical and non-clinical staff<br>Peer workers<br>Clinical supplies<br>Transportation<br>Training	None. In the data collection tool, the allocation by program area was directly identified.
Unspecified program area	
Utilities and external services<br>Equipment<br>Country office<br>Headquarters<br>Startup	Weights based on staff time allocation among the seven program area# were used to allocate these costs among the 7 program areas

Notes: LINKAGES program areas: 1) engage KPs in population size estimation, mapping, and program planning; 2) KP empowerment and engagement; 3) structural interventions; 4) peer outreach; 5) clinical services; 6) program management; and 7) monitoring and data use

$PAW_{PA} = \frac{\sum_{i=1}^{n} TD_{i,k,PA}}{\sum_{PA=1}^{7} \sum_{i=1}^{n} TD_{i,k,PA}}$

where PAW =program area staff time weight, k=drop-in center, i=individual, PA=program area, TD=time allocation

Supplement to: Opuni M, Figueroa JL, Sanchez-Morales JE, et al. The cost of providing comprehensive HIV services to key populations: an analysis of the LINKAGES program in Kenya and Malawi. *Glob Health Sci Pract*. 2023;11(3):e2200538. https://doi.org/10.9745/GHSP-D-22-00538

Supplement Table S4. Number of contacts and costs per contact by service level, KP served, and by country

	KENYA FY 2019				MALAWI FY 2019	
KP SERVED	FSW/MSW/ MSM	FSW	MSW	MSM	FSW	MSM/TGW
NUMBER OF DICS (*n*)	14	11	3	2	11	4
NUMBER OF CONTACTS						
Mean (SD)	5,667 (9,651)	6,622 (15,044)	12,157 (18,395)	875 (641)	1,300 (426)	865 (312)
Range	954–1,328	580–51,823	806–33,382	422–1,328	725– 2,159	570–1,274
Median (IQR)	2,768 (2,786)	2.367 (2,829)	2,284 (32,576)	875 (906)	1,190 (734)	808 (477)
**COST PER CONTACT**						
ABOVE SERVICE LEVEL						
Mean (SD)	31 (20)	49 (40)	41 (44)	113 (82)	142 (43)	214 (72)
Range	2–76	1–124	2–89	54–171	78–224	132–295
Median (IQR)	26 (25)	30 (67)	32 (87)	113 (117)	142 (78)	214 (115)
IP/DIC LEVEL						
Mean (SD)	52 (26)	57 (34)	57 (44)	180 (186)	98 (18)	120 (30)
Range	5–94	7–120	6 –87	49–311	79–130	80–151
Median (IQR)	53 (29)	48 (59)	77 (81)	180 (263)	91 (36)	124 (37)
TOTAL						
Mean (SD)	100 (49)	128 (78)	110 (88)	336 (308)	225 (59)	344 (98)
Range	11–186	13–239	10–175	118–554	180–361	222–455
Median (IQR)	53 (11)	96 (138)	145 (165)	336 (436)	241 (93)	350 (144)

Notes: FY 2019 refers to U.S. Government fiscal year from October 1, 2018 to September 30, 2019. *DIC* drop-in-center, *n* number of drop-in centers, *IP* implementing partner, *IQR* inter-quartile range, *SD* standard deviation, *FSW* female sex worker, *MSW* male sex worker, *MSM* men who have sex with men, *TGW* transgender women. Mean (SD), range, and median (IQR) of contacts and costs per contact calculated across all drop-in centers serving a given KP population in the sample for each country.

Supplement to: Opuni M, Figueroa JL, Sanchez-Morales JE, et al. The cost of providing comprehensive HIV services to key populations: an analysis of the LINKAGES program in Kenya and Malawi. *Glob Health Sci Pract*. 2023;11(3):e2200538. https://doi.org/10.9745/GHSP-D-22-00538

Supplement Table S5. Breakdown of capital supply costs by type of supply at the IP/DIC level

Clinical supplies	KENYA FY 2019		MALAWI FY 2019	
	USD 2019	%	USD 2019	%
PEP drugs	11,717	0.7	-	-
PrEP drugs	38,076	2.3	1,290	0.5
HIV tests	91,222	5.6	15,049	5.7
ART drugs	498,986	30.3	98,455	37.6
STI supplies[¶]	3,468	0.2	13,628	5.2
SRH supplies[#]	6,075	0.4	10,465	4.0
MSV supplies[†]	3,365	0.2	1,869	0.7
Condoms & lubricants[§]	991,799	60.3	121,341	46.3
Total IP/DIC	1,644,707	100.0	262,096	100.0

Notes: FY 2019 refers to U.S. Government fiscal year from October 1, 2018 to September 30, 2019. *IP* implementing partner, *DIC*, drop-in-center, *PEP* Post-Exposure Prophylaxis, *PrEP*, Pre-Exposure Prophylaxis, *ART*, Antiretroviral therapy, *STI* Sexually transmitted infections, *SRH*, sexual and reproductive health; *MSV*, management of sexual violence. [¶]*STI supplies* include antibiotics, antivirals, antifungals and antiparasitics, STIs tests. [#] *SRH supplies* include combined oral contraceptive pills, progestin-only pills, patches, implants, Depo injections, emergency pills, intrauterine device, and other barrier methods. [†]*MSV supplies* include emergency pills, PEP drugs, HIV tests. [§]*Condoms & lubricants* include male and female condoms, lubricants.
